# Creating Ferromagnetic Insulating La_0.9_Ba_0.1_MnO_3_ Thin Films by Tuning Lateral Coherence Length

**DOI:** 10.1021/acsami.1c00607

**Published:** 2021-02-15

**Authors:** Chao Yun, Weiwei Li, Xingyao Gao, Hongyi Dou, Tuhin Maity, Xing Sun, Rui Wu, Yuxuan Peng, Jinbo Yang, Haiyan Wang, Judith L. MacManus-Driscoll

**Affiliations:** † Department of Materials Science and Metallurgy, University of Cambridge, 27 Charles Babbage Road, Cambridge CB3 0FS, United Kingdom; ‡ Materials Engineering, Purdue University, West Lafayette, Indiana 47907, United States; § State Key Laboratory for Mesoscopic Physics, School of Physics, Peking University, Beijing 100871, China; ∥ School of Physics, Indian Institute of Science Education and Research Thiruvananthapuram, Thiruvananthapuram, Kerala 695551, India

**Keywords:** lateral coherence length, ferromagnetic insulators, lightly doped manganite, vertically aligned nanocomposites, double exchange coupling

## Abstract

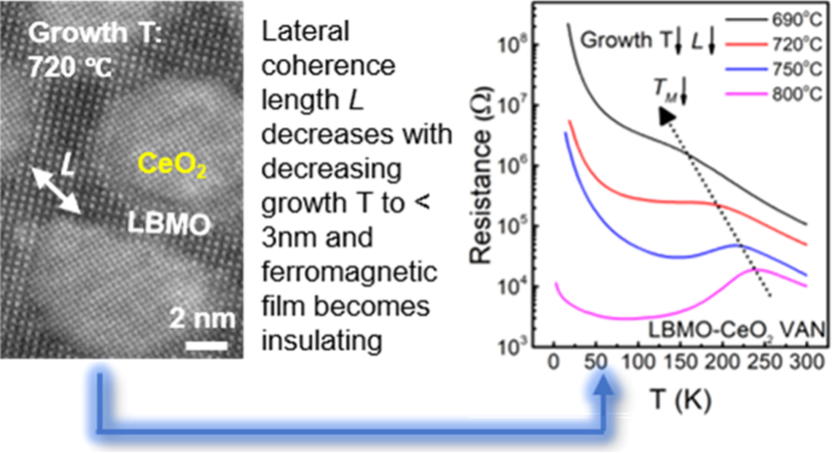

In this work, heteroepitaxial vertically aligned nanocomposite (VAN) La_0.9_Ba_0.1_MnO_3_ (LBMO)-CeO_2_ films are engineered to produce ferromagnetic insulating (FMI) films. From combined X-ray photoelectron spectroscopy, X-ray diffraction, and electron microscopy, the elimination of the insulator–metal (I–M) transition is shown to result from the creation of very small lateral coherence lengths (with the corresponding lateral size ∼ 3 nm (∼7 u.c.)) in the LBMO matrix, achieved by engineering a high density of CeO_2_ nanocolumns in the matrix. The small lateral coherence length leads to a shift in the valence band maximum and reduction of the double exchange (DE) coupling. There is no “dead layer” effect at the smallest achieved lateral coherence length of ∼3 nm. The FMI behavior obtained by lateral dimensional tuning is independent of substrate interactions, thus intrinsic to the film itself and hence not related to film thickness. The unique properties of VAN films give the possibility for multilayer spintronic devices that can be made without interface degradation effects between the layers.

## 1 Introduction

Vertically aligned nanocomposite (VAN) thin films have attracted significant attention^1,2^ due to their ability to three-dimensionally (3D) strain tune the physical properties of numerous functional systems, leading to improved ferroelectric, ferromagnetic, superconducting, and other functional properties.^3−6^ Furthermore, the electronic properties of vertical interfaces can be controlled to have either higher or lower conduction, depending on the materials used.^7−10^

It is widely known that film thickness is critical to the physical properties in standard epitaxial films of strongly correlated perovskite oxides. Indeed, several studies have been conducted on strongly correlated La_1–*x*_A*_x_*MnO_3_, where A = Ca, Sr, Ba. These materials are of particular interest because their physical properties are very sensitive to structural/compositional modifications, especially when the doping ratio is low (*x* < 0.2).^11^ When the film thickness is below ∼10 u.c., lattice-orbital-spin-charge degrees of freedom are strongly modified and so are the film properties. At the interface, “emergent” properties can be induced that are drastically different from either bulk or thick plain films and beyond the interfacial region, other “dead layer” effects come into play.^12−15^ The critical thickness below which the physical properties undergo drastic change is termed the “dimensional crossover”^16^ thickness. It can also be termed a *vertical* coherence length. In this low-thickness regime, in addition to modification of degrees of freedom from substrate interactions, strain-relieving defects from substrate–film lattice mismatch also come into play, which complicate the understanding of low-dimensional effects.^17−20^

The phase diagram of La_1–*x*_A*_x_*MnO_3_ covers almost all of the spectrum of critical functionalities for spintronic and multiferroic devices, ranging from ferromagnetic metals (which act as spin injection, detection layers in spintronics) to ferromagnetic insulators (which can be used in spin filters or in magnetoelectric devices). Within the lanthanum manganite family, La_1–*x*_Ba*_x_*MnO_3_ has the highest Curie temperature (*T*_c_) and is ferromagnetic insulating in the low-doped region. For example, the*T*_c_ of La_0.9_Ba_0.1_MnO_3_ is 185 K, while Sr- or Ca-doped counterparts have *T*_c_ < 150 K.^11,21,22^ La_0.9_Ba_0.1_MnO_3_ (LBMO) is particularly interesting for its FMI bulk properties, and we choose to focus on this composition in this work. It is well known that in thin films of LBMO, the FMI state cannot be easily achieved in films. Our recent study showed that it is possible to achieve this in films of thickness below ∼8 u.c. grown on SrTiO_3_. There, the SrTiO_3_ pins the octahedral rotations in the LBMO and decreases the Mn 3d e_g_ electronic bandwidth.^20,23^ While this effect is interesting, it is reliant on the underlying layer (whether substrate or another film) having unrotated, rigid, octahedra to prevent octahedral rotations in the LBMO film, and so is not widely applicable to device systems.

**Figure 1:**
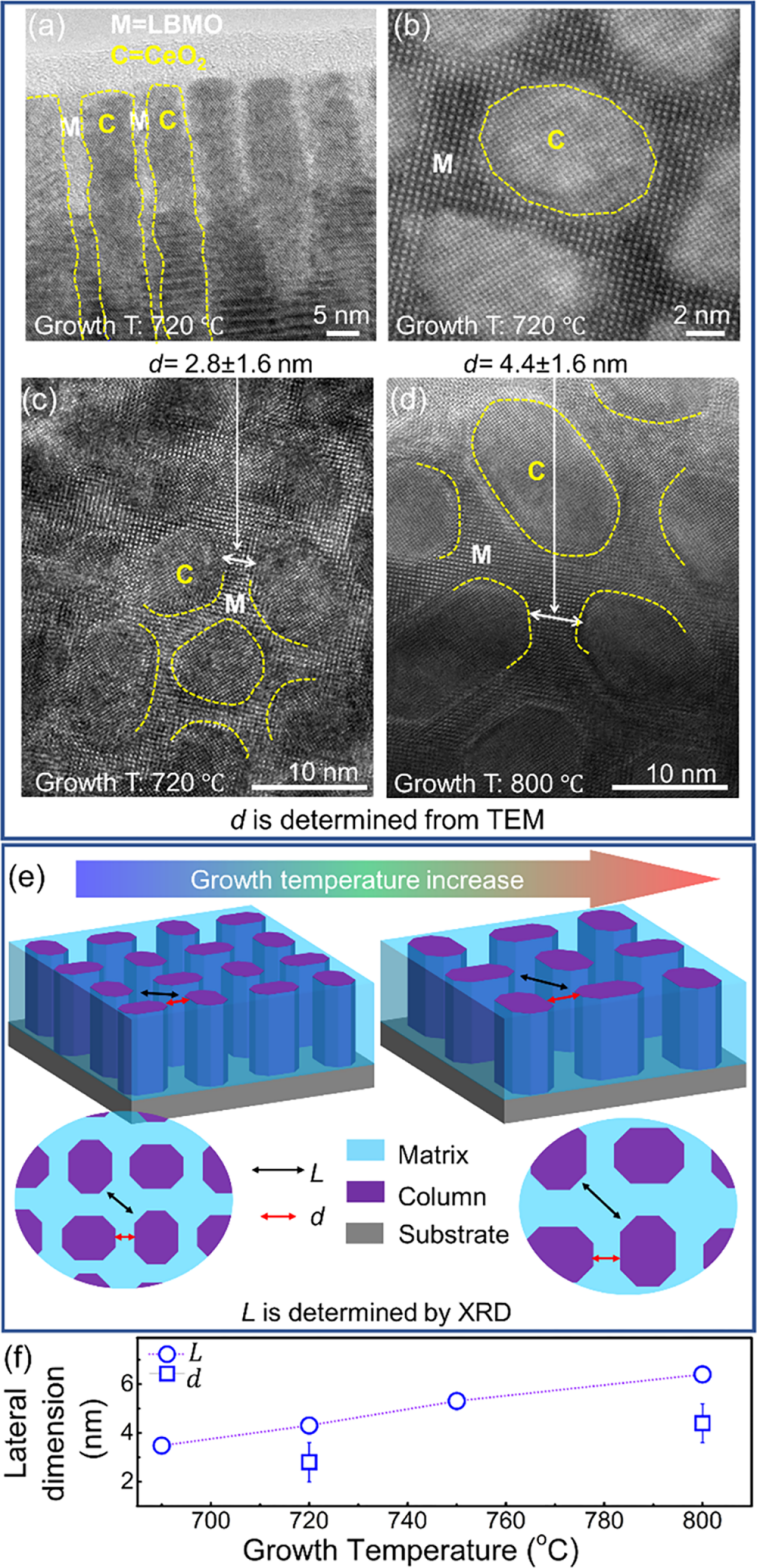
(a) Bright-field transmission electron microscopy (TEM) cross section of a 46 nm thick LBMO (M) -CeO_2_ (C) NC films grown at 720 °C. (b) Plan-view scanning TEM (STEM) image of the LBMO-CeO_2_ NC grown at 720 °C. Credit line: Adapted, reproduced in part with permission from ref (39). Copyright 2020 Royal Society of Chemistry. (c, d) Plan-view high-resolution TEM (HRTEM) images of the LBMO-CeO_2_ NC films grown at 720 and 800 °C, respectively. (e) Schematic diagram of VAN film showing *d* values, the shortest lengths in the matrix (*d*) compared to the lateral coherence lengths of the matrix (*L*) comprised of nanocolumns (purple) embedded inside a matrix (light blue). (f) *L* determined from Williamson–Hall analysis of ω rocking curves of X-ray diffraction (XRD) data, as discussed in Supporting Information S2 and S3, in comparison to *d* determined from TEM (examples of *d* shown in (c) and (d)).

Here, we control film properties without relying on the substrate. We do this using vertically aligned nanocomposite (VAN) films and explore the dependence of the physical properties of LBMO on coherence length. These films allow us to better understand interfacial effects in strongly correlated oxide films, so as to provide information about how to ultimately achieve controllable device properties. Here the coherence length is controlled laterally, rather than the standard vertically (via film thickness). VAN films are formed of a matrix phase of LBMO, with embedded self-assembled columns of a second phase that interdisperse in the matrix and break up the overall coherence of the matrix (Figures 1e and S2). *L* is the lateral “coherence length”, defined as the physically separated “mosaic block” average lateral dimension of the LBMO film between the columns.^24,25^ We also define *L*_column_ as the coherence length of the column phase (Figure S2). Since each “mosaic block” coherently scatters X-rays,^24^ the change of *L* can be precisely detected using X-ray characterization.

Crucially*, L* is different from the aforementioned *vertical* coherence length because it can be changed *independent* of substrate effects (namely, direct substrate interaction effects and strain effects), which the vertical effect cannot.

The benefits of VAN films for studying and exploiting lateral coherence length effects are:

1. The strain is much more uniform in VAN films than in plain films, especially when the film thickens. This is because strain in the VAN film above a certain thickness is controlled by the nanocolumns embedded in the film, rather than freestanding on the substrate. Hence, one can change *L*, while keeping a more uniform vertical strain.^4^ Also, since the film strain is dominated by the nanocolumns rather than by other film (or substrate) layers below the film, this may enable the desired functionalities of multilayer device structures to be attained.
2. *L* can readily be tuned in VAN films by controlling the distribution, size, and morphology of the columns.^2,26^ The self-assembly kinetics of film growth enable these dimensional features to be carefully engineered.^3,7,27^
3. Since the VAN vertical interface is self-assembled and has a much slower growth rate compared to the plain film interface, this enables high-quality vertical interfaces to be formed without any chemical reactions taking place at the interfaces.^18,28−31^

In a previous study, we showed that VAN LBMO films can be made much less conductive than plain films through changing film thickness.^39^ This study goes well beyond the previous work by achieving highly insulating films using a different approach. Hence, instead of changing film thickness, we tune *L*. In doing so, we traverse the “dimensional crossover” limit and eliminate deleterious effects resulting from substrate interactions, which has not been done before. More broadly, we open up a new and more flexible approach for engineering the physical properties of transition-metal oxides.

We show that when *L* is reduced (by reducing film growth temperature), the films switch from being ferromagnetic metallic (FMM) to ferromagnetic insulating (FMI) at *L* < 10 u.c. (4 nm). As we show later, this corresponds to a lateral size *d* of the LBMO from the nanopillar surface of <7 u.c. (3 nm). Accompanying this transition, we find a drastic shift of the valence band maximum (VBM) and decrease in the Mn 3d e_g_ density of states near *E*_F_, indicating a decrease in the Mn 3d e_g_ electron bandwidth. At the same time, *T*_c_ is not reduced compared to the bulk value, as is normally the case for reducing the vertical coherence length (plain film thickness). Our result holds strong promise for spintronic devices, where FMI films are required, better than substrate or underlayer control of the film properties as it is commonly the case for spintronic multilayer devices.^32^

## 2 Experimental Methods

Sample preparation: La_0.9_Ba_0.1_MnO_3_-CeO_2_ (molar ratio 1:1) nanocomposite films were grown on single crystalline SrTiO_3_ (001) substrates via a one-step process using pulsed laser deposition (PLD). The composite PLD target was prepared using a conventional solid-state sintering: stoichiometric and high-purity La_2_O_3_, Mn_2_O_3_, and BaO powders were mixed, grounded, and sintered at 900 °C for 40 h, and then reground and pelletized after mixing with CeO_2_, followed by an additional sintering at 1100 °C for 9 h. During deposition, the oxygen partial pressure was maintained at 0.2 mbar and growth temperature varied from 690 to 800 °C. A KrF excimer laser with a 248 nm wavelength was used. The repetition rate and laser fluency were 1 Hz and 1 J/cm^2^, respectively. After deposition, the sample was cooled down to room temperature under an oxygen pressure of 0.4 atm, with a cooling rate of 10 °C/min.

Sample characterization: The structure of the films was characterized with a Panalytical Empyrean high-resolution X-ray diffraction (XRD) system. The film thickness was controlled by the identical number of laser pulses during growth and was obtained by Laue fringes through XRD scans. Cross-sectional and plain-view images of the film were obtained by a high-resolution transmission electron microscope (HRTEM) FEI TALOS F200X at 200 kV equipped with ultrahigh-resolution high-angle annular dark-field detectors and a Super-X electron-dispersive X-ray spectrometer. The samples for the TEM analysis were obtained through mechanical grinding, dimpling, and a final ion milling step. SEM images of the VAN films were acquired using a Hitachi S-5200 SEM operated at 15 kV. The sample surfaces were coated with Ag prior to the detection to minimize the charging effects caused by insulating samples. Magnetic and transport property measurements were performed using a superconducting quantum interference device (SQUID) magnetometer (MPMS, Quantum Design) and a physical properties measurement system (PPMS, Quantum Design). Platinum electrodes were deposited by DC sputtering for standard four-probe characterization of the transport properties. X-ray photoelectron spectroscopy (XPS) was used to study the valence band of the films by a monochromatic Al Kα_1_ X-ray source (*h*ν = 1486.6 eV) using a SPECS PHOIBOS 150 electron energy analyzer with a total energy resolution of 500 meV. To prevent charging effects during the measurements, the samples were grown on (001) Nb-STO substrates, while all of the other samples were grown on undoped STO substrates. The Fermi level of the films was calibrated by a polycrystalline Au foil.

## 3 Results and Discussion

Four LBMO-CeO_2_ (molar ratio 1:1) VAN nanocomposite (defined as NC) films were grown using four different growth temperatures (690, 720, 750, and 800 °C). Three reference LBMO plain films (defined as PF, grown at 690, 720, and 800 °C) were also grown. The thickness of these films was ∼45 nm.

Figure S1 shows the XRD 2θ-ω scans of the NC films in comparison to a PF grown at 720 °C. The LBMO peaks of the NC films are all very close to or overlapped with the STO peaks, due to the very close lattice parameters (*a*_LBMOpc_ = 3.88–3.92 Å^33−38^ and *a*_STO_ = 3.905 Å). The thickness fringes that exist in all of the NC films near the STO (002) peak clearly indicate the existence of high-quality LBMO phase.

Figure 1 shows the electron micrograph images and schematic images of the NC films, where both *d* and *L* are also shown. *d* is defined as the average shortest LBMO distance between the columns and can be determined simply by inspection of planar TEM micrographs. We note here that while *L* is of interest to us, it is not possible to measure it directly owing to overlapping of the (00*l*) LBMO peaks with (00*l*) STO peaks (Figure S1). Hence, *L* is determined by extrapolation (Supporting Information S2 and Figure S2) from the measurement of *L*_column_ using ω rocking curves of X-ray diffraction, as described in detail in Supporting Information S3 and Figure S3.

Figure 1a,b shows the TEM cross-sectional and plan-view STEM images of the NC film grown at 720 °C, while Figure 1c,d shows HRTEM plan-view images and gives the measured *d* values of the NC films grown at 720 and 800 °C, respectively. In all images, clear phase separation and high-quality epitaxy is observed, with the CeO_2_ nanocolumns found to be evenly distributed in the LBMO matrix. The columns become more faceted with increasing growth temperature as the kinetics enable sufficient mobility of atoms to form lower-energy faces.^40^

Figure 1e shows a schematic diagram of the VAN film microstructure emphasizing how *d* differs slightly from *L*. Figures 1e and S2 also illustrate that the matrix and column dimensions *simultaneously* increase with growth temperature. Figure 1f shows how the calculated values of *L* from XRD and directly measured values of *d* change with growth temperature; *d* is *lower* than *L*, as expected because *d* is the shortest geometric LBMO distance between columns along the perpendicular direction, while *L* is an average dimension, which includes all lateral distances that radiate away from perpendicular distance from the columns, regardless of nonequal *d* existed in three dimensions. *L* is the physically more important distance in relation to the physical properties.

When the growth temperature increases from 720 to 800 °C, *L* (and *d*) increases from 3.48 (and 2.0–3.6) nm to 6.40 (and 3.6–5.2) nm, with *L* being around 25–45% larger than the average value of *d*. The increase in both these dimensions is expected based on the increase in diffusion coefficient with temperature. Hence, a thermally activated exponential dependence of *L* on 1/*T* very well fits the data, as shown in Figure S3b.(26)

The Williamson–Hall analysis of XRD rocking curves to calculate *L* (Figure S3a), the close fit of *L* to the measured *d* values for 720 and 800 °C (Figure 1f), as well as the very good fit of a nucleation and growth model to the *L* values (Figure S3b) confirm the *L* values determined for the four temperatures studied. We note that the ability to calculate *L* rather than measure it from TEM data is useful very broadly across other VAN systems, as it avoids the need to do time-consuming TEM. We also note that there have been many previous studies on VAN films showing growth temperature-dependent evolution of VAN dimensions, and physical properties, and our work is in broad agreement with the dimensional trends obtained previously.^41,42^

**Figure 2:**
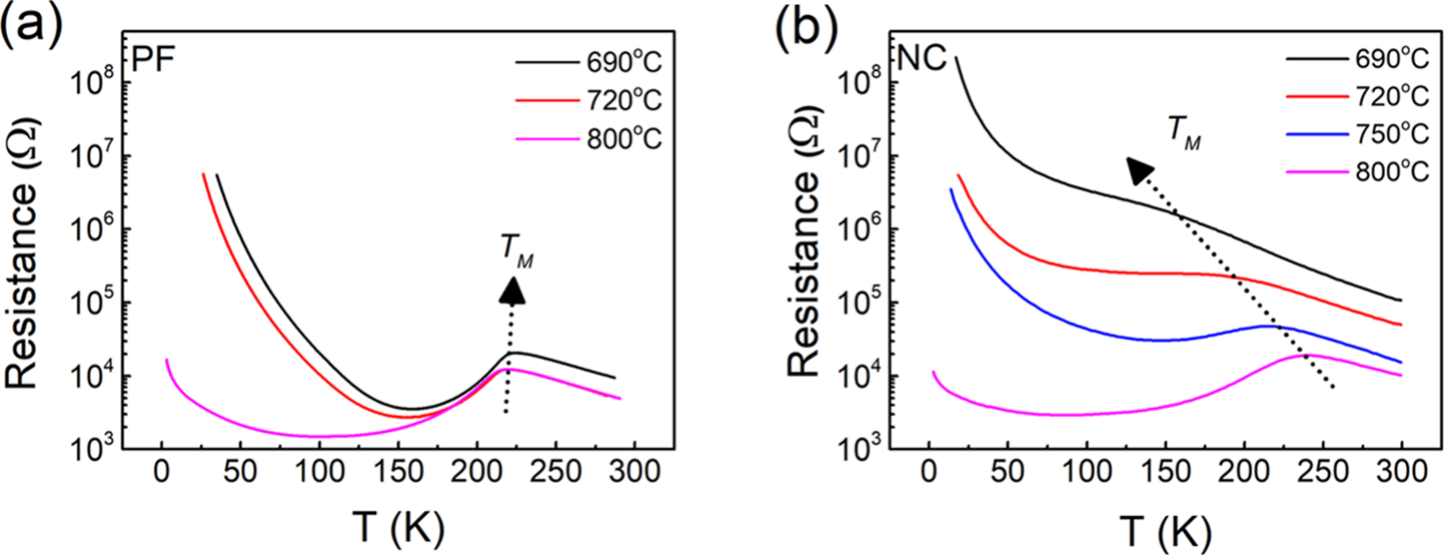
Growth temperature-dependent *R*–*T* curve for (a) the PF and (b) NC films. The films all have a thickness of ∼45 nm.

As shown in Figure 2a, all of the PFs show a clear insulator-to-metal (I–M) transition at around 221 K. This means that growth temperature has little influence on the transition temperature *T*_M_. In contrast, for the NC films, upon decreasing the growth temperature from 800 to 690 °C, the films change from ferromagnetic metal (FMM) to ferromagnetic insulating (FMI) behavior, as illustrated by the dashed arrow in Figure 2b. The I–M transition is gradually washed out as the temperature is decreased and disappears for the 690 °C-grown film, which is highly insulating throughout the whole temperature range. The room temperature/30 K resistance of the NC film grown at 690 °C is 2/4 orders of magnitude larger than the room temperature/30 K value of the NC film grown at 800 °C, i.e., 10^5^ Ω vs 10^4^ Ω at room temperature and 10^7^ vs 10^3^ Ω at 30 K.

As shown in Figure S4, for the PF films, the ferromagnetic-to-paramagnetic transition temperature *T*_c_ remains almost constant at around 213 K. In the NC films, as the growth temperature is decreased from 800 to 690 °C, *T*_c_ also decreases from 223 to 167 K. Here, *T*_c_ is determined at the temperature where d*M*/d*T* reaches the maximum. While the strain state of the LBMO did not change to a clearly measurable extent (from Figure S1), *L* approximately halved (Figure 1e and Table S1).

Figure S5 shows the magnetization vs. magnetic field loop for the NC films. All of the four samples exhibit typical ferromagnetic behavior. It is noted that the saturation magnetization (*Ms*) decreases from 441 emu/cm^3^ to 391, 355, and then to 280 emu/cm^3^ when the growth temperature decreases from 800 to 690 °C. This decrease in *Ms* could be due to the increase in interfacial area caused by the decrease in *L*.

The simultaneous tuning of *T*_c_ and *T*_M_ in the NC films indicates a strong modification of double exchange (DE) arising from modification of the electronic band structure. We now turn to understanding the origin of the drastic property tuning of NC films, i.e., PF. The change in the electronic band structure can be studied indirectly by measuring the Mn–O bond angle/length or the Mn 3d e_g_ orbital occupancy through global structural characterization (i.e., lattice parameters or *c*/*a* ratio). It can also be studied directly by in-depth probing of the electronic band structure. As already mentioned, there is a close overlap of the XRD peaks between LBMO and STO (Figure S1), and so the precise determination of LBMO lattice parameters is not possible. We therefore turn to X-ray photoelectron spectroscopy (XPS) to investigate the changes in the electronic structure of LBMO with growth temperature.

**Figure 3:**
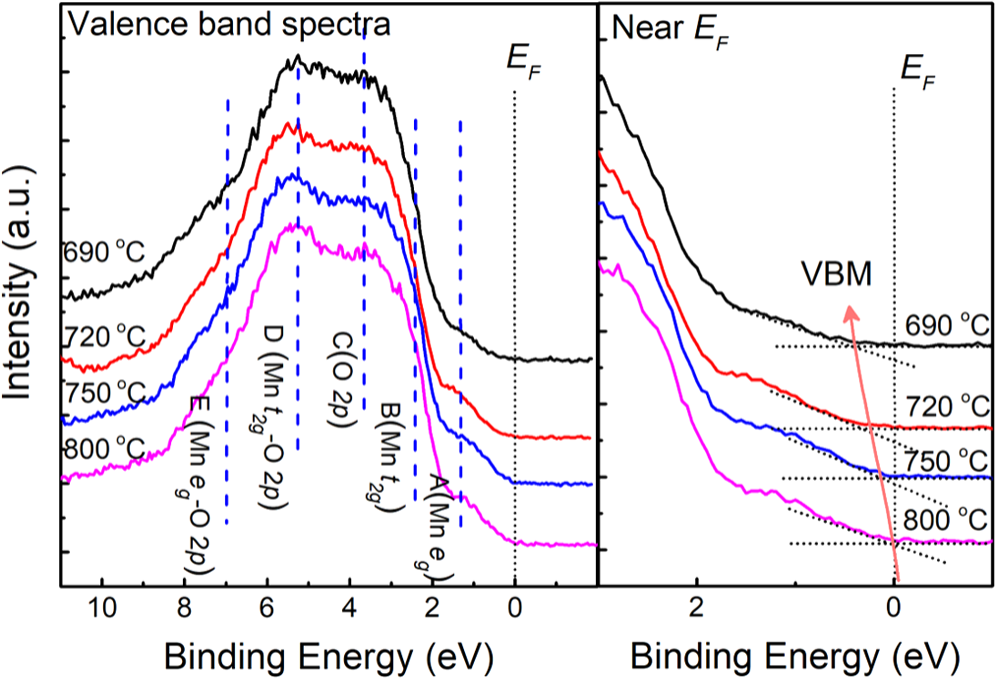
Left: XPS valence band spectra of the LBMO-CeO_2_ NC films grown at different temperatures. Right: XPS valence band spectra near the Fermi level (*E*_F_). The red line is a guide to the eye, showing the movement of the valence band maximum (VBM) with the change of growth temperature.

Figure 3 (left) shows the complete XPS valence band (VB) spectra of the NC films. As illustrated by the blue dashed lines, five structures can be identified as labeled.^43,44^ The Fermi level is illustrated by the black dotted lines. The valence band maximum (VBM) positions were determined by linear extrapolation of the leading edge of the valence band region to the extended baseline of the spectra,^23^ as shown in the near-*E*_F_ spectra (Figure 3, right). When the growth temperature is reduced from 800 to 690 °C, the VBM shifts toward higher binding energies (from 0.04 to 0.42 eV) and the e_g_ state of the Mn 3d orbital is well below *E*_F_, indicating that the films become more insulating.^23^ This is in good agreement with the observation of the change in transport and magnetic properties of the NC films (Figures 2 and S4), indicating an intrinsic change in the Mn 3d electronic band structure of LBMO.

**Figure 4:**
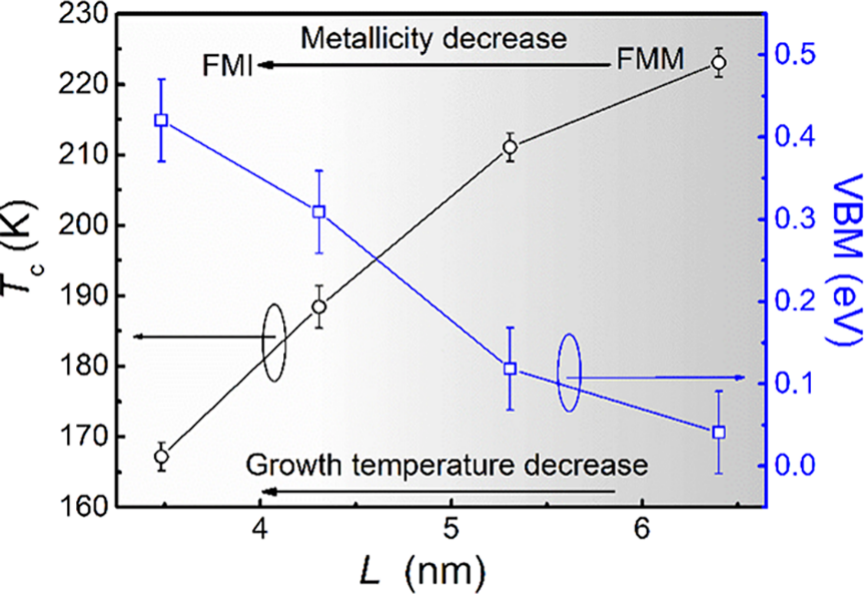
Relation between *T*_c_ and VBM to lateral coherence length, *L*. The depth of shading represents the extent of the metallicity, with more insulating behavior for smaller *L* values.

We now study how *L* controls the electronic properties (electrical resistivity and band structure). The VBM values from Figure 3, along with *T*_c_, are plotted versus *L* in Figure 4. The plot shows an inverse correlation between *T*_c_, metallicity, and *L*. As we explain below, the smaller *L* produces more insulating material by tuning the Mn 3d electronic structure (higher shift of the VBM observed).

Dimensional modulation of the electronic band structure and other physical properties has been reported previously in manganites, but the origin is controversial, with octahedral deformations, modification of the Mn–O bond length and orbital occupancies, nonstoichiometry, and phase separation being put forward.^12−15^ All of these modulations are correlated to the DE coupling.^11^ Here, our XPS results and their correlation to the physical properties are similar to the trend reported in the SrVO_3_^16^ films and plain LBMO films,^20^ where a metal-to-insulator transition was found upon decreasing the thin film thickness, correlated to a higher shift of the *3*d states of V or Mn located at *E*_F_ (i.e., a higher shift of VBM), decrease of density of states near *E*_F_, and reduction in the Mn 3d e_g_ one-electron bandwidth, *W*_a_. The latter results from the emergence of a “pseudo” band gap at *E*_F_ due to the absence of a density of e_g_ states when the coordination number of the 3d ions is reduced.^16^ A reduction in *W*_a_ and band gap is observed, which accounts for the tendency of insulating behavior.^20^

Here, in the LBMO-CeO_2_ VAN, when *L* is decreased, there are more uncoordinated Mn and La (or Ba) bonds blocked by CeO_2_ columns as the surface area-to-volume ratio and interface area both increase. The uncoordinated bonds lead to a reduction in density of e_g_ states near *E*_F_, which can result in a reduction in *W*_a_(16,20) and a reduction of the DE coupling hopping integral between adjacent Mn ions, *t*_*ij*_, as shown in eq 1(45)
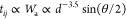
1where *d* is the Mn–O bond length. This explains the transition from FMM to FMI behavior and to the moderate *T*_c_ reduction.

In addition to creating more uncoordinated bonds, a smaller *L* means a larger LBMO/CeO_2_ interfacial area owing to denser CeO_2_ columns, which can also lead to greater control of the *op* strain state of LBMO by CeO_2_, i.e., on *c*_LBMO_, then influences the bond length *d* or preferential occupancy of *d*_3*z*^2^–*r*^2^_ orbitals,^45−48^ and hence *t*_*ij*_ (eq 1). This effect has been previously shown by in-depth studies on other VAN systems.^41,42^

As already noted, *c*_*LBMO*_ cannot be obtained accurately from XRD data owing to some overlap of LBMO peaks with the STO peaks. However, we observe a small left shift of the LBMO (003) peak of the 720 °C-grown sample compared to the 800 °C grown sample (illustrated in Figure S1a), indicating an increase in *c*_LBMO_ with decreasing growth temperature. This is consistent with a previous report.^49^ A further corroboration of increasing *c*_LBMO_ with decreasing temperature comes from the change of CeO_2_*op* lattice parameter. With decreasing growth temperature, c_CeO_2__ increases from 5.44 to 5.48 Å for 800 to 690 °C (see Figure S1a) corresponding to a 0.7% increase in *op* strain. Since the mechanically softer LBMO^3,50,51^ is vertically clamped by the stiffer CeO_2_ (*E*_CeO_2__ = 220–240 GPa^52,53^) with 2:3, 3:4, or 5:7 domain matching,^49,54^*c*_LBMO_ should also increase, which can lead to the increase in *d* or preferential occupancy of *d*_3*z*–*r*^2^_ orbitals;^45−48^ hence, *t*_*ij*_ will be reduced, another factor explaining the transition from FMM to FMI behavior.^55^ In contrast to the LBMO VAN films, we note that the LBMO PF films do not show a clear trend of *c*_LBMO_ with growth temperature, as evidenced by the LBMO (003) peak positions showing no clear shift (Figure S1b).

We note that apart from the modulation of DE coupling (the *T*_M_/*T*_c_), the overall resistance of the VAN films increases with decreasing growth temperature. This is well understood based on the reduced *L* value and increased interfacial area with the CeO_2_ and hence increased electronic scattering, which therefore induces a more rapid increase in resistivity than the decrease in ferromagnetic *T*_c_.

Finally, as mentioned above, the tuning of the DE coupling can also have a compositional origin. Since light Ce doping in LBMO has been found in our previous work in the LBMO-CeO_2_ NC grown at 720 °C,^39^ one possible compositional origin for the progressive change in *T*_c_ can be explained as a progressive change in the Mn^4+^/Mn^3+^ ratio caused by a change in the Ce doping content in the LBMO phase. This origin can be directly eliminated since the *T*_c_ evolution of the NCs crosses over that of the PFs and the NCs grown above 750 °C have higher *T*_c_’s than those of the reference PFs (Figure S4b), which cannot be explained by Ce doping. Also, since Ce^3+^ or Ce^4+^ has higher valences than Ba^2+^, Ce doping can indeed reduce the hole carrier concentration when doped into LBMO, and hence Ce doping should reduce the *T*_c_ value of LBMO instead of increasing it. As intermixing in VAN is always favored by a higher growth temperature,^56^ the postulated result is opposite to the result observed here. Therefore, even though light Ce doping of LBMO is deemed to exist in the NC films, Ce doping alone fails to explain the progressive tuning of *T*_c_ and metallicity with the change in growth temperature. Instead, a structural origin should be a more dominant cause, i.e., a change in the bandwidth irrelevant to chemical substitution, as suggested above.

**Figure 5:**
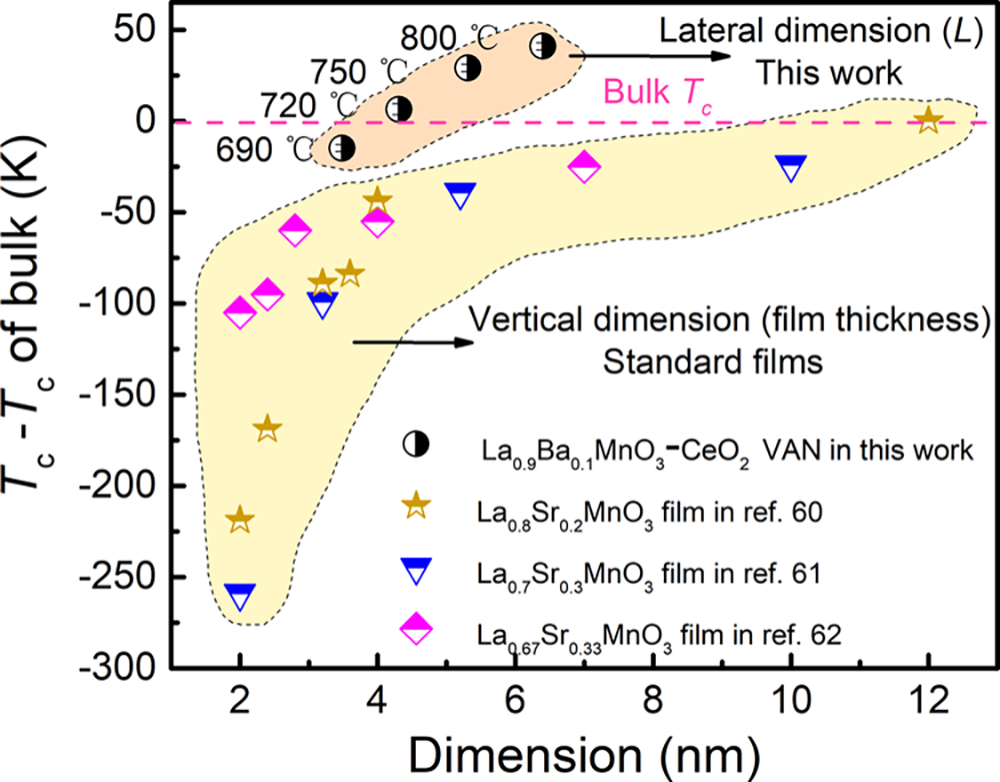
Comparison of *T*_c_ in LBMO-CeO_2_ VAN films, ultrathin manganite plain thin films with similar feature dimension, and respective bulk manganite.^60−62^ For plain films, vertical thickness is used, and for VAN films, the lateral coherence length (*L*) is used.

We now compare the influence of our measured lateral coherence length (*L*) effect on bulk *T*_c_ suppression with the vertical coherence length effect (different film thicknesses) from the literature for La_1–*x*_Sr*_x_*MnO_3_ (LSMO) films, *x* = 0.2–0.33 (Figure 5). For LSMO, there is a large body of data that allows a clear observation of suppressed *T*_c_ in films below about 12 nm by up to 50 K before the dead layer thickness is reached at about <3 nm (7 u.c.) when *T*_c_ drops more sharply. Compared to the vertical coherence length effect, our VAN films do not show a *T*_c_ suppression below the bulk value, except for the 690 °C-grown film with the smallest *L* of ∼3.5 nm (∼8.7 u.c). We note that this *L* value gives a *d* value of 1.9–2.6 nm (∼4.8 to 6.5 u.c., estimated based on the relative relationship of *d* and *L* in Figure 1f, i.e., *L* is 25–45% larger than *d*), which is more directly comparable to film thickness for the plain films (as it is the shortest distance from the interface). At 1.9–2.6 nm, this value is at the border of the “dead layer” zone. It is also worth noting that the VAN film is grown below the optimum temperature (>700 °C) for high crystalline perfection in a plain film^57−59^ and so a stronger *T*_c_ reduction would be expected considering the proximity to the “deal layer” thickness *and* the non-optimum growth temperature.

The different lateral and vertical behaviors can be attributed to the different nature of vertical interfaces in VAN films compared to the planar film/substrate interface in plain films. Reduced *T*_c_’s with film thickness, ultimately leading to a “dead layer” in plain films, originates from substrate strain and associated strong structural/compositional modifications.^61,63^ This “dead layer effect” is reduced here owing to both the more uniform vertical strain effect from the VAN columns and the more perfect atom-by-atom stacking along the vertical interfaces during the slow growth of the vertical interfaces.^18,26^

Overall, in VAN films, by engineering very low lateral coherence lengths, *L*, down to 8.7 u.c. (3.5 nm) in the film matrix, it has been possible to create FMI films with a relatively high *T*_c_ of 167 K. The control of the matrix by vertical interfaces as opposed to the substrate interfaces enables the high *T*_c_ to be maintained to lower *L* values.

On a final note, since VAN films enable FMI properties to be realized *intrinsically within* the film without domination of the underlying layers (e.g., from a substrate or another film), this opens up possibilities for new spintronic device concepts formed of multilayer VAN films.

## 4 Conclusions

We tuned the properties of LBMO from a ferromagnetic metal to a highly resistive ferromagnetic insulator using self-assembled LBMO-CeO_2_ VAN films. The control of lateral coherence length, *L*, led to a “dimensional crossover”, consistent with a modulation of the valence band maximum and density of e_g_ states near *E*_F_, tuning of the DE coupling, and thus tuning of *T*_c_ and metallicity. In contrast to VAN vertical thickness control, our new approach of lateral dimension tuning avoids clamping or strain effects from the substrate, thus eliminating deleterious interface interactions. Also, since *L* can be easily tuned in VAN structures simply made in a one-step process, VAN structures have the potential to offer more precise property control and simplicity of fabrication over top-down artificial designs, possibly opening up new pathways to novel spintronic devices.

## Supporting Information Available

Structural information of the LBMO-CeO_2_ NC and LBMO PF; geometric determination of *L* from *L*_column_; quantitative analysis of *L*_column_ (*L*_CeO_2__); influence of growth temperature on the ferromagnetic transition of the PF vs NC; and influence of growth temperature on the magnetic hysteresis loops of the NC (PDF)
