# Supplementary material for: Creating Ferromagnetic Insulating La0.9Ba0.1MnO3 Thin Films by Tuning Lateral Coherence Length
Source: ACS Appl Mater Interfaces. 2021 Feb 15;13(7):8863–70. doi: 10.1021/acsami.1c00607 (PMC8023513; doi:10.1021/acsami.1c00607)
Supplement: Supplementary file 1 — am1c00607_si_001.pdf [file am1c00607_si_001.pdf]

Supporting Information

# Creating Ferromagnetic insulating $La_{0.9}Ba_{0.1}MnO_3$ thin films by tuning lateral coherence length

*Chao Yun[a,c], Weiwei Li[a], Xingyao Gao[b], Hongyi Dou[b], Tuhin Maity[a,d], Xing Sun[b], Rui Wu[a], Yuxuan Peng[c], Jinbo Yang[c], Haiyan Wang[b] and Judith L. MacManus-Driscoll[a*]*

[a] Department of Materials Science and Metallurgy, University of Cambridge, 27 Charles Babbage Road, Cambridge, CB3 0FS, United Kingdom

[b] Materials Engineering, Purdue University, West Lafayette, IN, 47907, United States

[c] State Key Laboratory for Mesoscopic Physics, School of Physics, Peking University, Beijing 100871, China

[d] School of Physics, Indian Institute of Science Education and Research Thiruvananthapuram, Kerala, 695551, India

**Corresponding Author**

* Judith L. MacManus-Driscoll - Department of Materials Science and Metallurgy, University of Cambridge, 27 Charles Babbage Road, Cambridge, CB3 0FS, United Kingdom

ORCID: 0000-0003-4987-6620, Email: jld35@cam.ac.uk

## S1. Structural information of the LBMO-$CeO_2$ NC and PF.

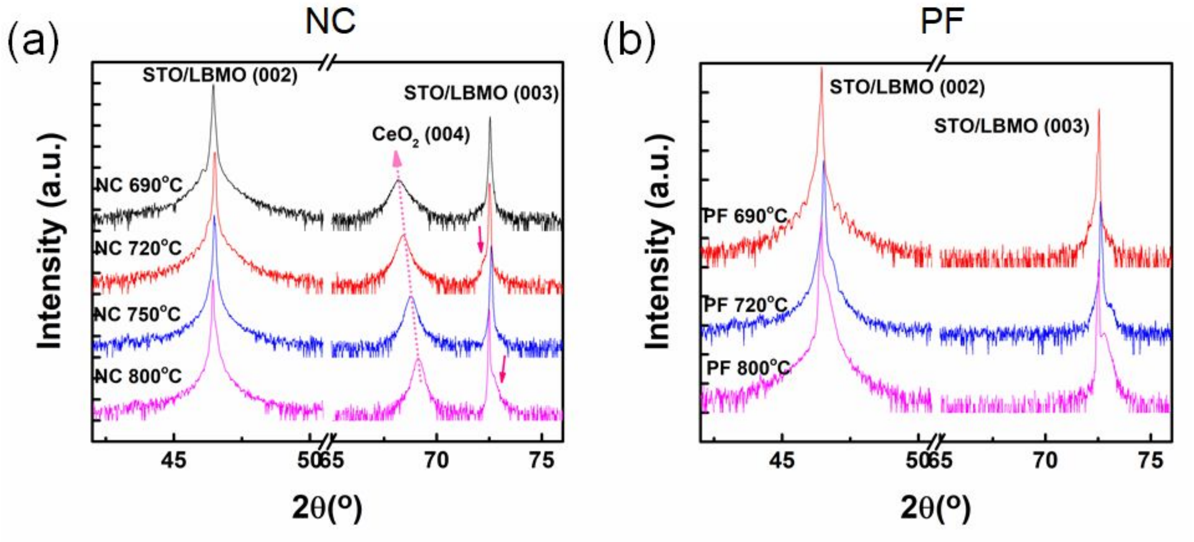


**Figure S1.** XRD $2\theta$-$\omega$ scans of the LBMO-$CeO_2$ NC films grown at different temperatures (a) in comparison with the LBMO PF films (b). Although the LBMO (00*l*) peaks of the NC films are obscured by the STO (00*1*) peaks, the $CeO_2$ (004) peak is distinct and not overlapped with any other peaks. It is also noted that the LBMO (00l) peaks of the PF films do not show a clear trend.

## S2. Geometric determination of *L* from $L_{column}$

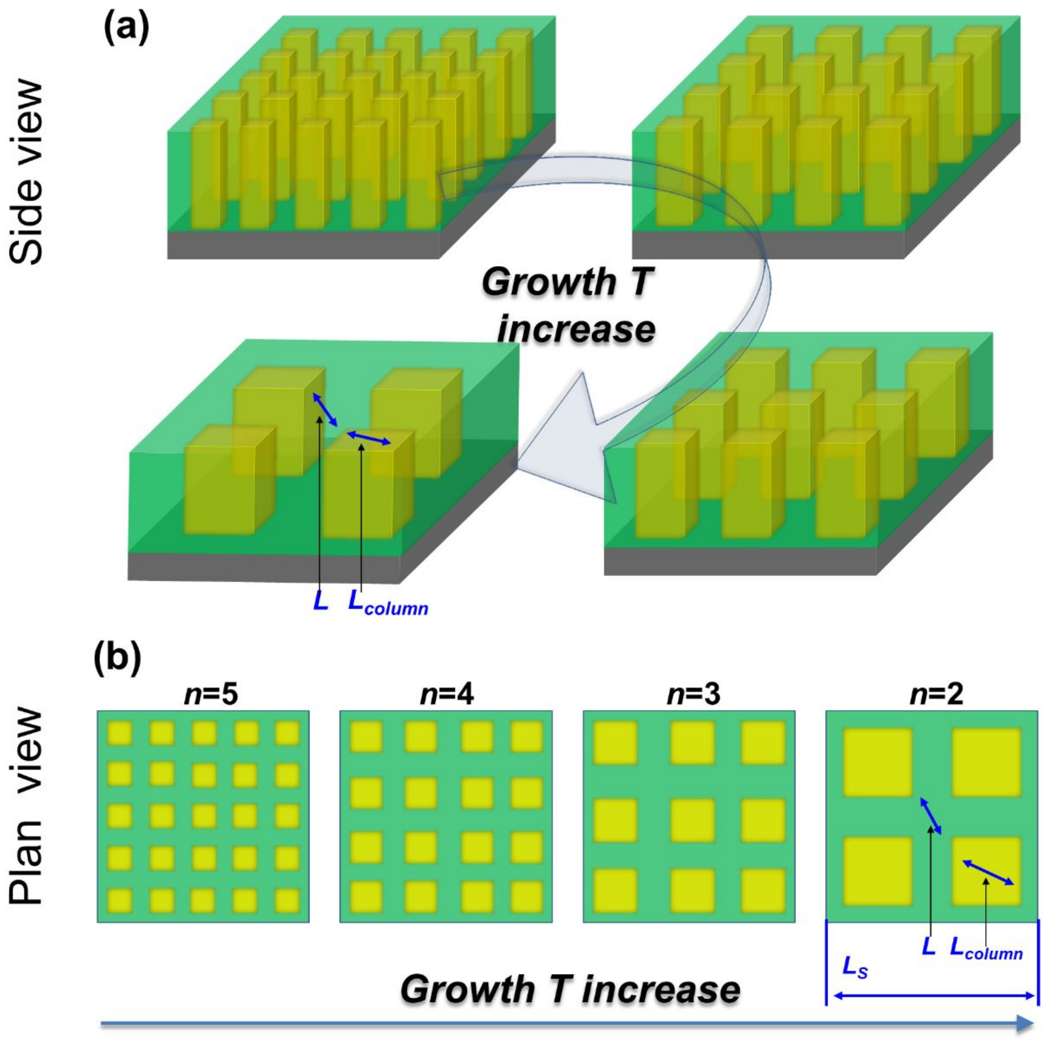


**Figure S2.** (a) Simplified 3D schematic illustration for the simultaneous change of lateral coherence length ($L$) and $L_{column}$ and with increasing growth temperature and (b) the corresponding plan view.

In the nucleation-and-growth mode, the change in the nanocolumn size is closely related to that of the matrix networks anchored among them. The increase in growth temperature results in a larger diffusion length of the constituent phases[1,2]. When the matrix: column volume ratio is fixed, the only way to respond to a larger diffusion length is to simultaneously expand the size of both phases. This is consistent with the trend shown in Figure 1: when the column size is larger, the distribution is more sparse, which leaves more space for the matrix in between.

In order to obtain a semi-quantitative relationship between the lateral coherence length ($L$) and $L_{column}$, we propose a simplified model for the simultaneous growth of matrix/column with temperature, which is illustrated in Figure S2a. For ease of calculation, we assume the columns have a square cross section and all the columns and matrix have equal size. In the case where the columns are evenly distributed, the relationship between $L$ and $L_{column}$ can be expressed as:

$$\frac{n}{\sqrt{2}}*(L + L_{column}) = L_S \tag{S-1}$$

$$(\frac{n}{\sqrt{2}}*L_{column})^2 = x*L_S^2 \tag{S-2}$$

where $x$ is the volume ratio of the column phase, $n$ is the number of nanocolumns per row along the edge of the sample, and $L_S$ is the sample width (assuming a square sample). Using Equations (S-1) and (S-2), $L_{column}$ and $L$ are given by:

$$L_{column} = \sqrt{x}*\frac{L_S}{n} \tag{S-3}$$

$$L = (1-\sqrt{x})*\frac{L_S}{n} \tag{S-4}$$

When the volume ratio $x$ is fixed, then:

$$\frac{L_{column}}{L} = \frac{\sqrt{x}}{1-\sqrt{x}} \tag{S-5}$$

In our LBMO-$CeO_2$ films, the LBMO:$CeO_2$ molar ratio is 1:1. Using the bulk density of $\rho_{CeO_2}$= 7.215 g/cm$^3$ [3] and $\rho_{LBMO}$= 6.6 g/cm$^3$ [4], this gives $x$ = 0.4. Therefore,

$$\frac{L_{column}}{L} = 1.72 \tag{S-6}$$

## S3. Quantitative analysis of $L_{column}$ ($L_{CeO_2}$)

$L_{column}$ ($L_{CeO_2}$) needs to be measured in order to enable $L$, the lateral coherence length to be determined in accordance with Equation S-6.

In VAN films consisting of a matrix with embedded nanocolumns, each nanocolumn blocks the continuity of the matrix. Both the column and matrix can be regarded as independent crystallites (called mosaic blocks) with a certain "coherence length"[5,6]. The lateral coherence length ($L$) of each phase is correlated to the mean extension along the finite-sized direction of each independent, unblocked crystal lattice regions. Since each "mosaic block" coherently scatters *X-rays*[5], $L$ can be precisely determined from *X-ray* characterization.

In an ideal case, a perfect crystal shows a sharp and strong $\omega$ rocking curve. However, in real cases, the full width at half maximum (FWHM) is broadened and broadening comes from the following factors[7]: (1) instrumental broadening due to limited equipment resolution, (2) limited lateral size of the phase, i.e. lateral coherence length ($L$), (3) tilt (and twist) of the unit cells ($\alpha$) and (4) microstrain ($\varepsilon$) due to the strain gradient from different depth levels or from the periphery to the center of the phase. In our experiment, the instrumental broadening due to resolution limit (0.006°) is much less compared to the peak width of the $\omega$ rocking curves (~1°) and can be ignored. In reciprocal space, broadening factors can be expressed as[7]:

$$\Delta s_L = \frac{1}{L} \quad \text{(S-7)}$$

$$\Delta s_\alpha = \alpha s \quad \text{(S-8)}$$

$$\Delta s_\varepsilon = 2\varepsilon s \quad \text{(S-9)}$$

where $s$ is the scattering vector and $\Delta s$ is the broadening in reciprocal space. For $\omega$ scans, their relationships to the real space parameters are defined as:

$$s = \frac{1}{d} = \frac{2\sin\theta}{\lambda} \quad \text{(S-10)}$$

$$\Delta s = \beta s \quad \text{(S-11)}$$

where $\beta$ is the integral breadth measured by $\omega$, which is obtained from dividing the peak area by the peak height.

In general, the sample broadening can be expressed as:

$$\beta_{sample} = \beta_{size} + \beta_{misorientation} + \beta_{microstrain} \quad \text{(S-12)}$$

when the peak shape is assumed to be Lorentzian.

$$\beta^2_{sample} = \beta^2_{size} + \beta^2_{misorientation} + \beta^2_{microstrain} \quad \text{(S-13)}$$

when the peak shape is assumed to be Gaussian.

For symmetric $\omega$ scans, there is no contribution from microstrain and twist to the peak broadening[8]. Therefore, Equations (S-12) and (S-13) can be simplified to Williamson-Hall (W-H) expressions[5,7,9,10] as below:

$$\Delta s = \alpha.s + \frac{1}{L} \text{ (for Lorentzian peak)} \quad \text{(S-14)}$$

$$\Delta s^2 = \alpha^2 s^2 + \frac{1}{L^2} \text{ (for Guassian peak)} \quad \text{(S-15)}$$

where $s = \frac{2sin\theta}{\lambda}$ is the scattering vector and $\Delta s = \beta\frac{2sin\theta}{\lambda}$ is the broadening in reciprocal space, $\beta$ is the integral breadth (peak area divided by the height) of the $\omega$ rocking curve, $\theta$ is the diffraction peak position in the corresponding $2\theta$-$\omega$ scan, $\alpha$ is the tilt angle, $L$ is the coherence length of the phase and $\lambda$ is the *X-ray* wavelength ($\lambda$=1.54 Å is used here).

Via linear fitting of the W-H plot (a plot of the $s$-$\Delta s$ for Lorentzian peak shape (or $s^2$-$\Delta s^2$ for Gaussian peak shape)) relationship as a function of the reflection order), the y coordinate intercept is taken to be the reciprocal of $L$ (or $L^2$)[7].

$L_{CeO_2}$ was determined by analyzing $\omega$ rocking curves of the $CeO_2$ peaks collected on all the NC samples. Assuming Pseudo-Voigt peak profile, the diffraction peaks can be fitted with either Lorentzian or Gaussian shapes depending on the shape factor (Lorentzian: Gaussian ratio, defined as a function of the FWHM/$\beta$ ratio)[7]. The W-H plots from Gaussian peak fitting, which gives the closest results to our experiment results, are shown in Figure S3a.

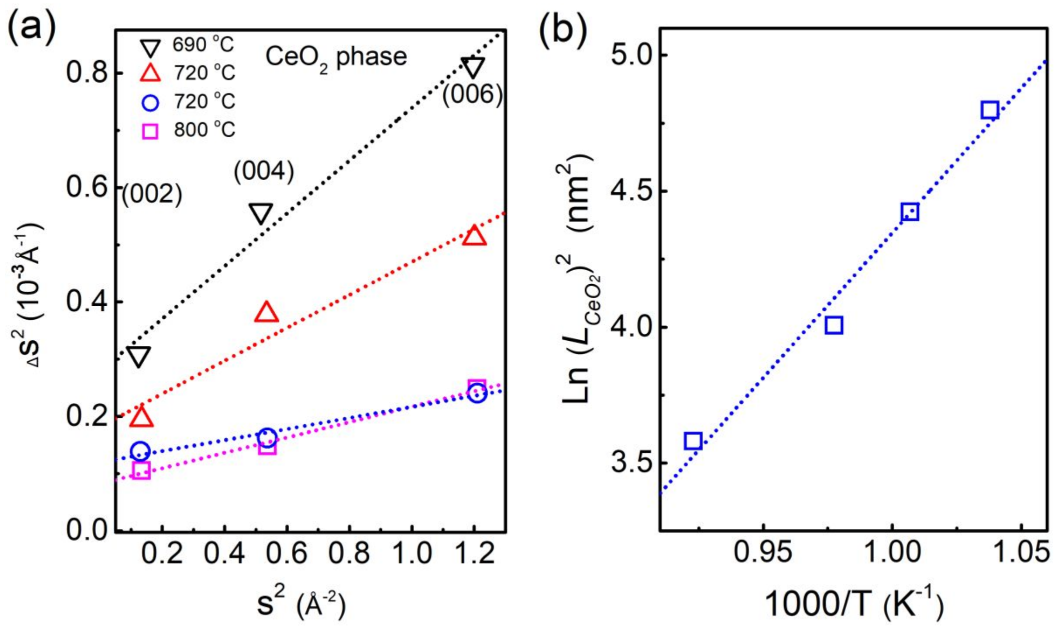


**Figure S3.** (a) Williamson-Hall plots for the $CeO_2$ nanocolumns in the LBMO-$CeO_2$ NC films grown at different temperatures. The rocking curve parameters were obtained from Gaussian peak fitting. (b) Growth temperature-dependent fitting of the calculated $L_{CeO_2}$ based on the nucleation and growth mode (linear fitting of $(L_{CeO_2})^2$ versus inverse temperature).

The extrapolated $CeO_2$ lateral coherence length, $L_{CeO_2}$, values for the different growth temperatures are shown in Table S1 below. As expected, $L_{CeO_2}$ increases with growth temperature, from ~5.99 to 11.01 nm, from 690 to 800 ℃. The calculated values of $L$ from $L_{column}$ ($L_{CeO_2}$) from Equation (S-6) are also shown in Table S1. The data is shown in the plot of Figure 1f.

**Table S1** $L$ calculated from $L_{column}$ ($L_{CeO_2}$)

| **Growth temperature (℃)** | **690** | **720** | **750** | **800** |
|---|---|---|---|---|
| $\boldsymbol{L_{column}}$ ($\boldsymbol{L_{CeO_2}}$) (nm) | 5.99 | 7.41 | 9.13 | 11.01 |
| $\boldsymbol{L}$ (nm) | 3.48 | 4.31 | 5.31 | 6.40 |

As shown in Figure S3b, a linear relationship is shown between $(L_{CeO_2})^2$ and inverse temperature ($\frac{1}{T}$) which is consistent with the formation of the columns in the matrix by nucleation-and-growth[2]:

$$x \approx 2\sqrt{D\tau} \qquad \text{(S-16)}$$

where $x$ is diffusion distance, and $\tau$ is the diffusion time, which is proportional to the laser repetition rate. $D$ is the diffusion coefficient[2] which follows the Arrhenius Equation:

$$D = D_0 * e^{-\frac{E_A}{kT}} \qquad \text{(S-17)}$$

where $D_0$ is a pre-exponential factor, $E_A$ is the activation energy, $k$ is the Boltzmann constant, and $T$ is the temperature. As the deposition time and laser repetition rate are constant, the diffusion time $\tau$ is constant, and thus the $L_{CeO_2}$ changes with the growth temperature in the following relationship: $(L_{CeO_2})^2 \propto \frac{1}{T}$.

**S4. Influence of growth temperature on the ferromagnetic transition of the PF vs. NC**

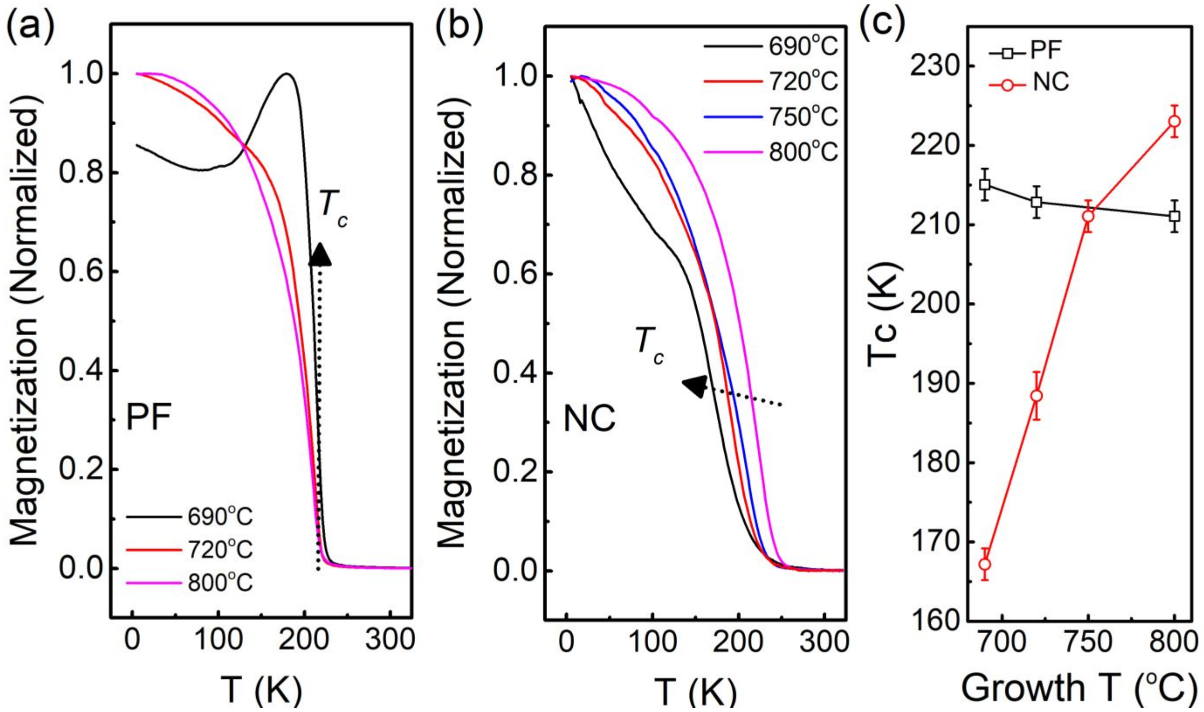


**Figure S4.** Growth temperature-dependent *M-T* curve for (a) PF and (b) NC films. The applied magnetic field was 200 Oe parallel to the substrate plane. (c) Comparison of $T_c$ between the PF and NC with variation in growth temperature. $T_c$ is defined as the temperature where *dM/dT* reaches the maximum.

## S5. Influence of growth temperature on the magnetic hysteresis loops of the NC films

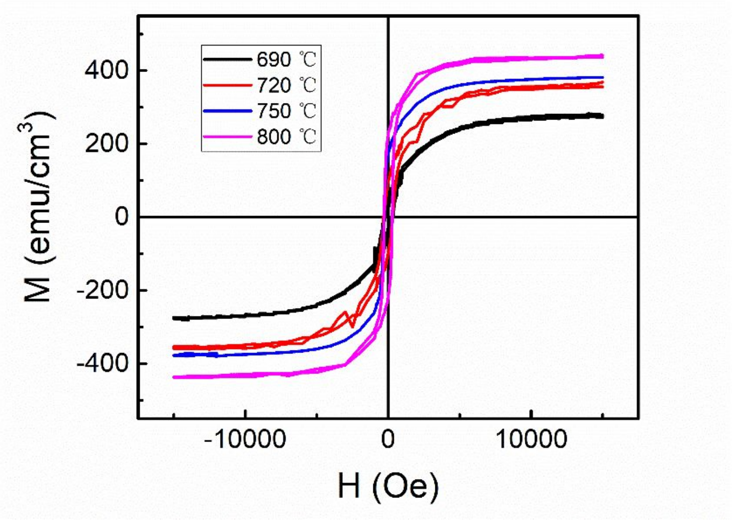


**Figure S5.** Influence of growth temperature on the magnetic hysteresis loops of the NC films.

## References


(1) Chen, A.; Hu, J.-M.; Lu, P.; Yang, T.; Zhang, W.; Li, L.; Ahmed, T.; Enriquez, E.; Weigand, M.; Su, Q.; Wang, H.; Zhu, J.-X.; MacManus-Driscoll, J. L.; Chen, L.-Q.; Yarotski, D.; Jia, Q. Role of Scaffold Network in Controlling Strain and Functionalities of Nanocomposite Films. *Sci. Adv.* **2016**, *2* (6), e1600245. https://doi.org/10.1126/sciadv.1600245.

(2) Chen, A.; Bi, Z.; Jia, Q.; MacManus-Driscoll, J. L.; Wang, H. Microstructure, Vertical Strain Control and Tunable Functionalities in Self-Assembled, Vertically Aligned Nanocomposite Thin Films. *Acta Mater.* **2013**, *61* (8), 2783–2792. https://doi.org/10.1016/j.actamat.2012.09.072.

(3) Https://En.Wikipedia.Org/Wiki/Cerium(IV)_oxide.

(4) Lee, H.-Y.; Liu, H.-J.; Hsu, C.-H.; Liang, Y.-C. Preparation of $La_{0.7}Sr_{0.3}MnO_3/LaNiO_3$ Magnetic Oxide Superlattice Structure by Rf Sputtering. *Thin Solid Films* **2006**, *494* (1–2), 325–329. https://doi.org/10.1016/j.tsf.2005.08.161.

(5) Liu, B.; Zhang, R.; Xie, Z. L.; Lu, H.; Liu, Q. J.; Zhang, Z.; Li, Y.; Xiu, X. Q.; Chen, P.; Han, P.; Gu, S. L.; Shi, Y.; Zheng, Y. D.; Schaff, W. J. Microstructure and Dislocation of Epitaxial InN Films Revealed by High Resolution X-Ray Diffraction. *J. Appl. Phys.* **2008**, *103* (2), 023504. https://doi.org/10.1063/1.2832753.

(6) Chierchia, R.; Böttcher, T.; Heinke, H.; Einfeldt, S.; Figge, S.; Hommel, D. Microstructure of Heteroepitaxial GaN Revealed by X-Ray Diffraction. *J. Appl. Phys.* **2003**, *93* (11), 8918–8925. https://doi.org/10.1063/1.1571217.

(7) Moram, M. A.; Vickers, M. E. X-Ray Diffraction of III-Nitrides. *Reports Prog. Phys.* **2009**, *72* (3), 036502. https://doi.org/10.1088/0034-4885/72/3/036502.

(8) Sangle, A. L. Nanostructured $SrTiO_3$ for Strongly Enhanced Tunable Microwave and Photocatalytic Performance, PhD Thesis, University of Cambridge, 2016.

(9) Chierchia, R.; Böttcher, T.; Heinke, H.; Einfeldt, S.; Figge, S.; Hommel, D. Microstructure of Heteroepitaxial GaN Revealed by X-Ray Diffraction. *J. Appl. Phys.* **2003**, *93* (11), 8918–8925. https://doi.org/10.1063/1.1571217.

(10) Vickers, M. E.; Kappers, M. J.; Datta, R.; McAleese, C.; Smeeton, T. M.; Rayment, F. D. G.; Humphreys, C. J. In-Plane Imperfections in GaN Studied by x-Ray Diffraction. *J. Phys. D. Appl. Phys.* **2005**, *38* (10A), A99–A104. https://doi.org/10.1088/0022-3727/38/10A/019.
